# Elimination of virus-like particles reduces protein aggregation and extends replicative lifespan in *Saccharomyces cerevisiae*

**DOI:** 10.1073/pnas.2313538121

**Published:** 2024-03-25

**Authors:** K. L. Schneider, X. Hao, K. S. Keuenhof, L. L. Berglund, A. Fischbach, D. Ahmadpour, S. Chawla, P. Gómez, J. L. Höög, P. O. Widlund, T. Nyström

**Affiliations:** ^a^Department of Microbiology and Immunology, Institute for Biomedicine, Sahlgrenska Academy, Centre for Ageing and Health—AgeCap, University of Gothenburg, Gothenburg 40530, Sweden; ^b^Department for Chemistry and Molecular Biology, University of Gothenburg, Gothenburg 41390, Sweden

**Keywords:** proteostasis, protein aggregation, mitochondria, aging, virus-like particles

## Abstract

A major consequence of aging and stress, in yeast to humans, is an increased accumulation of protein aggregates at distinct sites within the cells. Using genetic screens, immunoelectron microscopy, and three-dimensional modeling in our efforts to elucidate the importance of aggregate annexation, we found that most aggregates in yeast accumulate near the surface of mitochondria. Further, we show that virus-like particles (VLPs), which are part of the retrotransposition cycle of Ty elements, are markedly enriched in these sites of protein aggregation. RNA interference-mediated silencing of Ty expression perturbed aggregate sequestration to mitochondria, reduced overall protein aggregation, mitigated toxicity of a Huntington’s disease model, and expanded the replicative lifespan of yeast in a partially Hsp104-dependent manner. The results are in line with recent data demonstrating that VLPs might act as aging factors in mammals, including humans, and extend these findings by linking VLPs to a toxic accumulation of protein aggregates and raising the possibility that they might negatively influence neurological disease progression.

## Significance

Virus-like particles (VLPs) are the products of endogenous retroelements in the yeast, *Saccharomyces cerevisiae*. Retroelement propagation is linked to aging, but the mechanisms of their effects are still emerging. We show that VLPs are markedly enriched in sites of protein aggregation. RNAi (RNA interference)-mediated silencing of retroelement expression perturbed aggregate sequestration to mitochondria, reduced overall protein aggregation, mitigated toxicity of a Huntington’s disease model, and expanded replicative lifespan in a partially Hsp104-dependent manner. These findings link VLPs to a toxic accumulation of protein aggregates and raise the possibility that they might negatively influence neurological disease progression. Our findings therefore reveal consequences of VLP expression and further our understanding of how aging, age-related diseases, and protein quality control function are linked.

Protein folding diseases such as Alzheimer’s and Parkinson’s disease are characterized by the accumulation of aberrant, aggregated proteins. There is also evidence of mitochondrial dysfunction in these age-associated neurodegenerative diseases (1–3). While the relationship between protein aggregation and mitochondrial function remains to be fully understood, work in budding yeast suggests that the conserved spatial protein quality control (PQC) machinery may be a potential link. Heat-induced protein aggregates were found to be sequestered and deposited at mitochondria, a process required for proper asymmetric inheritance of protein aggregates (4). Additionally, aggregates in the proximity of mitochondria were shown to be cleared out faster than those in distal cytosolic locations (5) and imported into mitochondria for degradation (6). It is mechanistically not clear how this mitochondrial localization of protein deposits may be beneficial for spatial PQC or other cellular functions during stress.

In addition to the aggregate localization at mitochondria, other sites where aberrant proteins are deposited have been defined, namely the JUNQ (JUxta Nuclear Quality control compartment) near the cytosolic side of the nuclear membrane, the INQ (IntraNuclear Quality control compartment) in the nucleus near the nuclear membrane, and the IPOD (Insoluble Protein Deposit) at the vacuole (7–11). Importantly, JUNQ and IPOD were recently observed surrounded by mitochondrial cages (12), highlighting that inclusions localize near several organelles simultaneously. The spatial PQC machinery relies on a multitude of sorting factors, including molecular chaperones, which facilitate the deposition of misfolded proteins/aggregates at certain sites (9, 13). Differences between misfolded protein species have also been found to impact sorting to these sites, which was most apparent when comparing amyloidogenic with amorphously aggregating proteins: Amyloidogenic proteins such as the Huntingtin misfolding protein were targeted exclusively to the IPOD, while most ubiquitinated misfolded proteins were sorted to the JUNQ/INQ (7). The IPOD may even exist as two distinct compartments depending on the protein species, which could explain why some neurodegenerative disease proteins are observed mostly at the vacuole, while endogenous misfolding protein reporters localize to mitochondria (4, 7, 14, 15). Importantly, yeast cells undergo large organellar changes during heat stress (16), which may influence spatial PQC due to the confinement of aggregates to organelles and other cellular structures such as actin (4, 7, 13, 17).

Here, using immunoelectron microscopy and 3D modeling and a genome-wide high-content imaging screen, we identified proteins required for localization of aggregates to the surface of mitochondria, including Clu1 and proteins forming virus-like particles (VLPs). The *CLU1* gene encodes a translation initiation factor required for normal mitochondrial morphology, whereas the Ty retrotransposons encode Gag, the main structural component of VLPs and Gag-Pol, which additionally contains elements required for retrotransposition and its regulation (18). Ty elements comprise ca. 3.5% of the yeast genome (S288C) and exist in five families named Ty1 to Ty5 (19, 20). We found that fully formed Ty VLPs colocalize with aggregates in areas of ribosome exclusion at the vicinity of mitochondria. Further, we found that Ty VLPs play a hitherto unknown role in PQC using RNAi (RNA interference) to silence Ty1-5 retroelement expression. In Ty-silenced strains, spatial PQC during heat shock (HS) is improved, and cells appear less susceptible to the toxicity of the Huntingtin disease protein. We also find that silencing of Ty retroelement expression extends replicative lifespan in a partially Hsp104-dependent manner, further demonstrating a link between retroelement expression and maintenance of proteostasis during aging. In contrast, deleting *CLU1* had no discernible effect on PQC indicating that the effects seen in Ty-silenced cells are not entirely due to a reduced accumulation of aggregates to mitochondria. A link between VLPs and PQC is interesting in view of recent data demonstrating that VLPs might act as aging factors in mammals, including humans (21).

## Results

### Protein Aggregates Induced by HS Form Areas of Ribosome Exclusion Near Mitochondria.

**Fig. 1.**
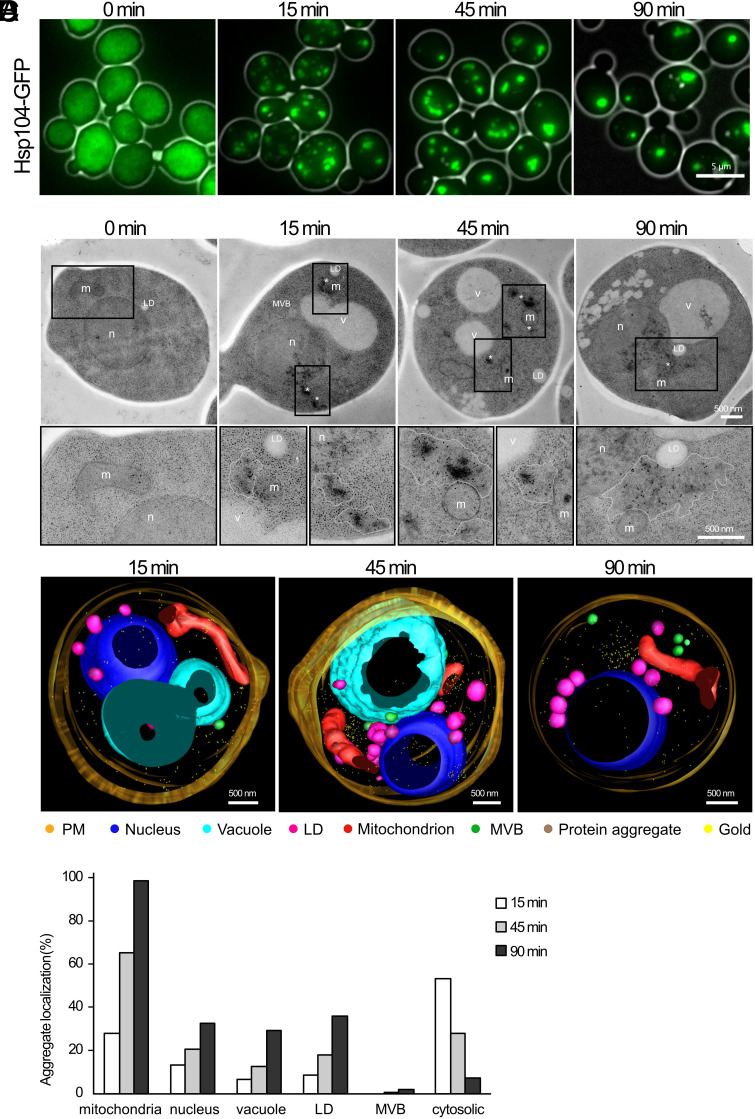
Heat-induced Hsp104-GFP-containing protein aggregates are often in proximity to mitochondria. (*A*) Fluorescent images of live cells expressing Hsp104-GFP subjected to continuous HS at 38 °C for indicated times. (Scale bar, 5 µm.) (*B*) Immuno-EM with anti-GFP of cells grown as in (*A*). Nucleus (n), vacuole (v), mitochondria (m), lipid droplet (LD), multivesicular body (MVB), asterisk = gold enrichment. Outline = ribosome free area. Black rectangles indicate the image area that was enlarged. Contrast and brightness were changed in enlarged images for better visualization of gold particles. (*C*) 3D models of Hsp104-GFP expressing cells during continuous HS at 38 °C at indicated time points. Seventy-nanometer-thin serial sections were immunogold labeled against GFP and modeled using IMOD. The legend depicts the color for each modeled structure. (*D*) Quantification of Hsp104-GFP aggregate localization. No aggregates were found before HS. A total of 100 cells were analyzed per time point. Aggregates localized to several structures were scored for all relevant categories, n: 15 min = 185, 45 min = 145, 90 min = 82 aggregates.

The response of the spatial PQC machinery to HS can be visualized using a GFP-tagged version of the protein disaggregase, Hsp104 (8, 22, 23). During exponential growth, Hsp104-GFP is distributed evenly throughout the cell, but when the temperature is raised, many small aggregates (Q-bodies) become visible in the cell at early time points, which then coalesce into fewer and larger inclusions with time, namely JUNQ/INQ and IPOD (Fig. 1*A*). To get a more detailed view of this process, we performed the same continuous heat stress experiment, except samples at each time point were instead prepared for electron microscopy followed by immunogold labeling with GFP antibodies to detect Hsp104-GFP (Fig. 1*B*). Gold particles were often located in areas of high electron density and in surrounding areas of the cytoplasm devoid of ribosomes. Analysis of gold labeling densities confirmed that these areas of ribosome exclusion are the location of heat-induced protein aggregates (*SI Appendix*, Fig. S1*A*). These areas were often located near mitochondria, vacuoles, nuclei, and lipid droplets (Fig. 1*B*). To visualize the cellular environment surrounding the protein aggregates, two serial thin-section reconstructions were generated for each time point of HS and modeled in 3D (Fig. 1*C*). The protein aggregate localizations, as determined by immunogold localization of Hsp104-GFP, in relation to mitochondria, nucleus, vacuole, lipid droplets, and multivesicular bodies were quantified (Fig. 1*D*). Aggregates that did not appear in proximity of any of these structures were assigned the category “cytosolic” and those that were proximal to several structures simultaneously were scored for all relevant categories. The data for each time point confirmed that small aggregates at early time points arose mainly in the cytosol and gradually localized near mitochondria (Fig. 1*D*). This general localization can also be seen by light microscopy using the mitochondrial marker Tom70-mRuby2 (*SI Appendix*, Fig. S1*B*) in agreement with previous reports (4, 5, 24). Aggregates were also localized, although less often, near several other organelles, including the nucleus, vacuole, and lipid droplets. Next to no protein aggregates were found near multivesicular bodies. To test whether this spatial localization of aggregates of endogenous misfolding proteins near mitochondria is a general phenomenon, we repeated the continuous HS experiment using Tom70-mRuby2 and each of one of the model misfolding proteins guk1-7-GFP, gus1-3-GFP, and pro3-1-GFP (5, 25–27). These readily misfolding temperature-sensitive proteins were previously found to behave similarly to Hsp104-GFP during heat stress and mostly colocalized with the disaggregase (27). Heat-induced aggregates of all three misfolding proteins formed inclusions near mitochondria to a high extent, indicating that the deposition of aggregates near mitochondria is a general phenomenon (*SI Appendix*, Fig. S1*C*).

### High-Content Imaging Screens of gus1-3-GFP and Hsp104-GFP Reveal Differential Requirements for Aggregate Localization to Mitochondria.

**Fig. 2.**
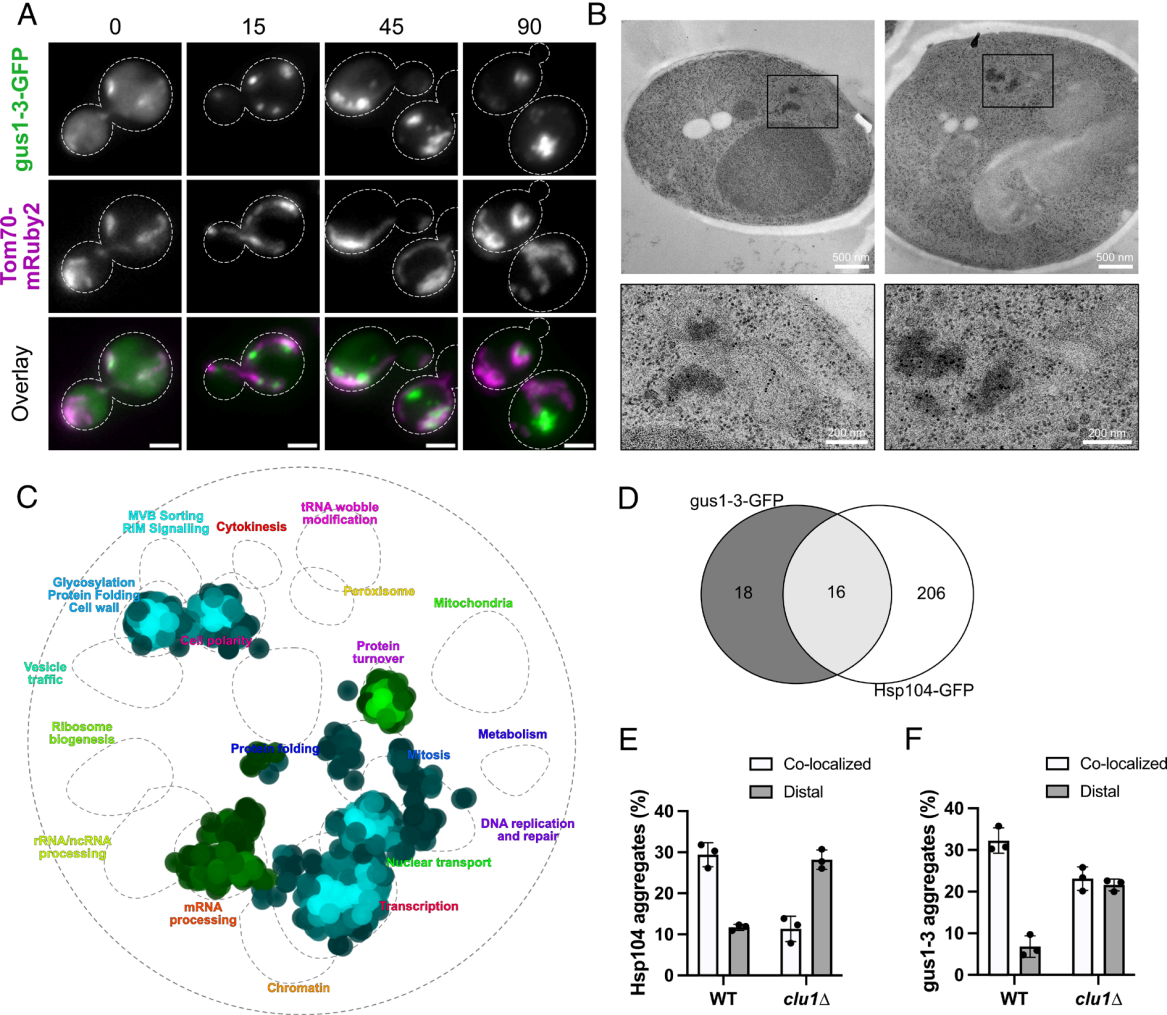
Factors sorting aggregates to mitochondria depend on the misfolded protein species. (*A*) LM of gus1-3-GFP and Tom70-mRuby2 expressing cells showing localization of aggregates to mitochondria at indicated time points (min) during continuous HS at 38 °C. (Scale bar, 2 µm.) (*B*) Immuno-EM with anti-GFP of cells expressing gus1-3-GFP gus1-3-mCherry at 38 °C 30 min HS. Black rectangles indicate the image area that was enlarged. Contrast and brightness were adjusted in enlarged images for better visualization. (*C*) SAFE analysis enrichments of hits among ts mutant collection for gus1-3-GFP (green) and Hsp104-GFP (blue), cutoff 15% deviation from control cells, visualized with thecellmap.org at *P* ≤ 0.05. (*D*) Venn diagram depicting overlap of mutants identified for both protein aggregation reporters and number of individual hits. (*E*) Quantification of Hsp104-GFP aggregate localization relative to mitochondria in cells exposed to 38 °C HS for 60 min and imaged with fluorescent microscopy. Aggregates of the different reporters were scored as colocalized, proximal, or distal to mitochondria, category proximal not shown for simplicity. (*F*) Quantification as described in E for gus1-3-GFP expressing cells after 60 min at 38 °C.

Aggregate localization to mitochondria has been shown to be important for three PQC processes: mother-biased retention of aggregates during cell division (4), aggregate clearance (5), and a mechanism that facilitates degradation of misfolded proteins in mitochondria (6). Because these studies relied on candidate-based approaches, we sought to identify more factors involved in this process using an unbiased screen. The misfolding model protein gus1-3-GFP was chosen as it showed the strongest colocalization with mitochondria tagged with Tom70-mRuby2 (*SI Appendix*, Fig. S1*C* and Fig. 2 *A* and *B*).

The strains for the screen were constructed using standard synthetic genetic array methodology (28) by mating the query strain expressing gus1-3-GFP and Tom70-mRuby2 with the yeast deletion and temperature-sensitive mutant collections. Cells were grown in 96 well-plates, heat shocked, visualized by high-content microscopy, and automatically analyzed with a software module that applied separate masks to aggregates and mitochondria and scored proximal and total protein aggregates. Among the deletion collection, no hits were identified with at least a 30% decrease of aggregate colocalization compared to the control, which was a cutoff we defined based on the random association simulation published by Zhou et al. (4). A 30% deviation from WT is substantial for this colocalization phenotype, considering the space that aggregates and mitochondria occupy in the cell. A less stringent cutoff (5% decrease) yielded 40 deletion mutants (*SI Appendix*, Table S2). The top hit among the deletion collection screen for gus1-3-GFP, *mdm12*Δ, was also identified as a factor required to deposit Hsp104-GFP aggregates at mitochondria (4), which is a validation of our approach and suggests that Mdm12 function is required for proper aggregate deposition of various protein species. An additional screen of the temperature-sensitive mutant collection revealed 38 hits/34 ORFs at a 15% cutoff, which we employed to be more stringent, including *myo2-14* (*SI Appendix*, Table S3). This agrees well with previous identification of a different Myo2 mutant, *myo2-16*, as important for Hsp104-GFP labeled aggregate localization to mitochondria [*SI Appendix*, Fig. S2*A*, (5)]. The hits were subjected to GO term analysis and enrichments were found for the proteasome, spliceosome, and processes related to their respective functions (*SI Appendix*, Table S4). SAFE analysis, which categorized the hits according to genetic profile interaction similarity (29), supported these enrichments (Fig. 2*C*). To determine whether the identified genes are specific to the misfolding protein gus1-3-GFP, we performed the same screen at a reduced scale for Hsp104-GFP using only the ts allele collection, since hits from this collection generally displayed a stronger phenotype than those in the deletion collection. For Hsp104-GFP, we identified 264 alleles/222 ORFs with a disruption of aggregate deposition at mitochondria (cutoff 15%), which were enriched in GO terms similar to the functions found with SAFE (*SI Appendix*, Tables S5 and S6 and Fig. 2*C*). Only 16 ts mutants showed mislocalization of aggregates of both reporters, demonstrating that even though endogenous misfolding proteins and gus1-3-GFP aggregates were sequestered at the same PQC site, they may rely, in part, on distinct factors to reach it (Fig. 2 *C* and *D*). In fact, we tested a previously established mutant of mitochondrial Hsp104-GFP deposition (4), *fis1*∆, and a mutant with dysfunctional mitochondrial organization, *mdm10*∆, for disruption of this pathway for gus1-3-GFP aggregates. Interestingly, while both mutants have defects in mitochondrial morphology, neither deletion affected localization of gus1-3-GFP aggregates to mitochondria (*SI Appendix*, Fig. S2*B*). Subjecting the mutants of the ts allele collection that were detected in both gus1-3-GFP and Hsp104-GFP screens to GO term analysis did not reveal any common enrichments of GO groups. We were thus unable to pinpoint commonalities within the pathway machineries. Mislocalization of aggregates did not correlate with the number of protein aggregates per cell for either protein species (*SI Appendix*, Fig. S2 *C* and *D*, linear regression gus1-3 R^2^ = 0.26, Hsp104 R^2^ = 0.19). For example, most mutants found for gus1-3-GFP of the enriched category RNA splicing have functional inclusion formation but show a defect in localization to mitochondria. In contrast, proteasomal mutants show defects in both inclusion formation and deposition at mitochondria. Therefore, mislocalization of aggregates distal from mitochondria cannot be solely attributed to mere overload of aggregates due to impaired inclusion formation.

### Partial Disruption of Inclusion Formation at Mitochondria Does Not Impair Proteostasis.

Based on our gus1-3-GFP screen data, we selected *clu1*∆ among the stronger hits as a model strain to monitor potential proteostasis defects caused by disrupted mitochondrial deposition of heat-induced aggregates as it retained functional inclusion formation. *CLU1* encodes an RNA-binding eukaryotic translation initiation factor three subunit of unknown biological function. Deletion of the gene does not cause growth defects, but cells display a clustered mitochondria phenotype, while remaining respiration-competent (30, 31). Additionally, lines of evidence connect Clu1 to mitochondrial function and neurodegenerative disease (32–34). We reconstructed the mutant in BY4741 cells and found that *clu1*∆ cells were significantly impaired, statistically, in gus1-3-GFP inclusion formation (*SI Appendix*, Fig. S2*E*); however, these defects were very modest. We manually confirmed the screen result, i.e., that the lack of Clu1 caused mislocalization of endogenous protein aggregates and gus1-3 upon heat stress (Fig. 2 *E* and *F*) and we were able to mostly rescue this phenotype by reintroducing the gene expressed under its native promoter into a secondary location in the genome (*SI Appendix*, Fig. S2 *F* and *G*). To test the relevance of Clu1 for proteostasis in a broader sense, we exposed *CLU1* overexpression and deletion strains to elevated temperature offering either glucose or glycerol as carbon source but observed no striking effects on growth (*SI Appendix*, Fig. S2 *H* and *I*). Likewise, no toxicity was detected for *clu1*∆ cells on plates containing azetidine-2-carboxylic acid, a proline analog that induces global protein misfolding (35) or expressing the misfolding protein gus1-3-GFP (*SI Appendix*, Fig. S2*J*).

### Misfolding Proteins and VLPs Colocalize Near Mitochondria during Mild HS.

**Fig. 3.**
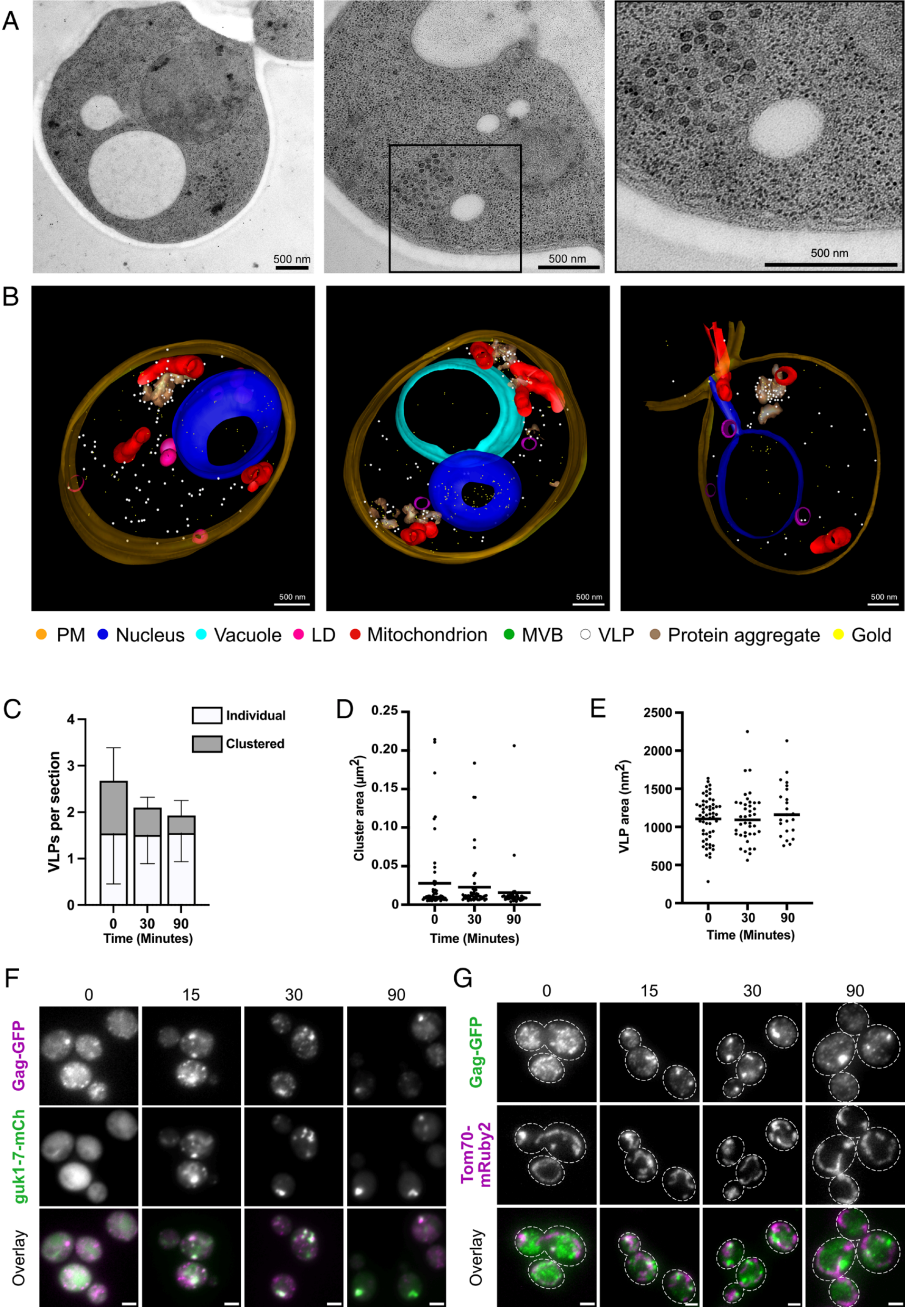
Heat-induced guk1-7-GFP protein aggregates are often in proximity to mitochondria and VLPs. (*A*) Immuno-EM with anti-GFP of cells expressing guk1-7-GFP at 38 °C 30 min HS. The black rectangle indicates the image area that was enlarged. Contrast and brightness were adjusted in enlarged images for better visualization. (*B*) XY view of 3D models of guk1-7-GFP expressing cells subjected to 30 min of 38 °C HS. Fourteen serial sections of 70 nm thickness each were immunolabeled against GFP and modeled using IMOD. The legend depicts the color for each modeled structure. (*C*) Quantification of clustered (≥2) and individual VLPs per 70-nm section using EM of Hsp104-GFP expressing cells during continuous 38 °C HS at indicated time points. Number of sections analyzed: 0 min 115, 30 min 111, and 90 min 120. (*D*) Quantification of VLP cluster size in EM of Hsp104-GFP expressing cells during continuous 38 °C HS at indicated time points. The line indicates mean. Number of clusters analyzed as in Table 1. (*E*) Quantification of individual VLP size in EM of Hsp104-GFP expressing cells during continuous 38 °C HS at indicated time points. The size was manually measured as spherical areas in IMOD. The line indicates mean. (*F*) LM of guk1-7-mCherry and Gag1-GFP expressing cells at indicated time points (min) during continuous HS at 38 °C. (Scale bar, 2 µm.) (*G*) LM of Tom70-mRuby2 and Gag1-GFP expressing cells at indicated time points (min) during continuous HS at 38 °C. (Scale bar, 2 µm.)

Localization of aggregates near mitochondria could only be reduced up to at most ~20% in our screens, as can be observed with *clu1*∆ (Fig. 2 *E* and *F*). However, even in wild-type cells, aggregates were often near, but not directly at mitochondria. We therefore looked for other cellular structures in the vicinity of protein aggregates that could contribute to aggregate localization and/or possibly account for the distinct aggregate deposition of various protein species using electron microscopy. Clusters of small, round structures were seen in proximity to aggregates of the ts misfolding model protein guk1-7-GFP (detected using immunogold labeling) at intermediate time points of mild HS in electron micrographs (Fig. 3 *A* and *B*). We identified these structures as VLPs (compare to micrographs in, e.g., refs. 36 and 37).

We applied continuous mild HS to Hsp104-GFP-containing cells and quantified different VLP characteristics with EM from three separate experiments (Fig. 3 *C*–*E* and Table 1). VLPs were observed individually as well as in clusters. The number of VLPs, VLP cluster size, and the number of VLPs per cluster decreased over the time course (Table 1 and Fig. 3 *C* and *D*), while the number of clusters per section, individual VLPs, and VLP size did not vary significantly (Table 1 and Fig. 3*E*). This overall decrease in VLP number could be due to degradation as we observed an initial decrease in Gag (VLP capsid protein) levels using an anti-Gag antibody in an identical time course (*SI Appendix*, Fig. S3*A*). However, we also observed partial recovery of Gag levels but without a simultaneous increase of VLPs in the EM, which suggests that VLP assembly lags behind Gag production.

**Table 1.** Analysis of VLPs during mild heat stress using EM

	0 min	30 min	90 min
VLPs per cluster (average)	7.08	5.41	3.11
Clusters per section (average)	0.18	0.14	0.11
Number of clusters analyzed	58	53	43

The table shows VLP properties during continuous HS at 38 °C sampled at the indicated time points. Total sections (each of a different cell) analyzed: 0 min 321, 30 min 329, and 90 min 347.

To further characterize VLP behavior, we visualized VLPs by tagging the Gag1 protein, the main structural protein of VLPs, with GFP [compare to micrographs in, e.g., ref. 38, *SI Appendix*, Fig. S3*B*]. Gag1-GFP represented ~19% of the total Gag levels we detected (*SI Appendix*, Fig. S3*C*). Though the fusion is expressed via the constitutive GPD promoter, the overall increase in Gag levels was modest compared to WT cells (*SI Appendix*, Fig. S3*D*). The Gag1-GFP construct was validated as a reporter for VLPs by performing immunofluorescence using a direct antibody against Ty Gag (*SI Appendix*, Fig. S3*E*). Before HS, Gag1-GFP appears as minuscule foci as well as a few larger foci throughout the cell, which likely represent individual and clustered VLPs observed by EM (Fig. 3*C*). Upon heat stress, larger Gag1-GFP foci appeared along with the minuscule foci. We found that guk1-7-mCherry aggregates and Hsp104-mRuby2 aggregates colocalized to a high extent with Gag1-GFP foci at different time points during continuous HS (Fig. 3*F* and *SI Appendix*, Fig. S3*F*). Gag1-GFP were also found to be proximal to mitochondria during HS (Fig. 3*G*), as predicted from the localization of protein aggregates to mitochondria (*SI Appendix*, Fig. S3*G*) and the colocalization of Gag1-GFP and aggregates.

### Protein Aggregates and VLPs Colocalize in a Heat Stress–Specific Manner Separate from RNP Granules.

We searched for proteins involved in retrotransposition among reported Hsp104 physical interactors enriched upon heat stress [(39), Table 2] and found the Ty Gag proteins, providing biochemical support for VLP interaction with aggregates. We also identified the P-body [processing body, a type of ribonucleoprotein (RNP) granule] component Dhh1 as a physical interactor of Hsp104. Ty3 VLPs have been shown to associate with components of P-bodies and P-body mutants show altered Ty3 VLP localization and morphogenesis (40). Genomic screens revealed that lack of P-body components *pat1*∆ and *lsm1*∆ affects Ty1 retrotransposition (38, 41), further suggesting a link between P-bodies and several Ty family proteins. However, Ty1/Gag foci are distinct from P-bodies (38, 42), in contrast to Ty3 foci. A relationship between P-bodies and stress granules (SG) (a type of RNP granule) to known PQC compartments, such as those found in INQ/JUNQ, has been explored and it was shown that these structures are distinct but also that functional P-body/SG formation appears important for proteostasis maintenance (43).

**Table 2.** Protein interactors of interest of Hsp104 identified as enriched upon HS by mass spectrometry

Gene name	Description	Fold enrichment at 38 °C
HSP104	HS protein 104	85.9
DHH1	ATP-dependent RNA helicase Dhh1	7.0
TY1B-MR1	Transposon Ty1-MR1 Gag-Pol polyprotein	3.8
TY1B-PR1	Transposon Ty1-PR1 Gag-Pol polyprotein	2.3
TY1B-JR2	Transposon Ty1-JR2 Gag-Pol polyprotein	2.5

Data was extracted from ref. 39. The complete table can be found in *SI Appendix*, Table S7. The third column shows the fold enrichment of detected peptides at 38 °C.

To determine the relative cellular location of VLPs and P-bodies or SGs, we subjected cells to both mild HS and the more severe conditions commonly used to visualize RNP granule formation (*SI Appendix*, Fig. S3*H*). Conditions used in our previous colocalizations of VLPs, protein aggregates, and mitochondria did not induce typical P-body formation, which we monitored using GFP-tagged Dhh1 (44). Upon nutrient removal, Dhh1 foci became visible but did not colocalize with Gag1-mCherry or with guk1-7-mCherry, which remained mainly soluble. Severe HS induced substantial aggregation of guk1-7, Gag1 and the SG marker Pub1-GFP (45). guk1-7- and Pub1 aggregates did not overlap entirely, while Gag1 and Pub1 showed clear colocalization. While numerous aggregates of guk1-7 and Gag1 may colocalize with SG during severe HS, these RNP granules did not form under mild heat stress conditions. These data suggest that formation of guk1-7 aggregates and VLP clusters and their colocalization occur independently of visible RNP granule formation and that the compartments of interest are predominantly separate from RNP granules. It is therefore possible that Hsp104 association with Dhh1 during HS occurs in a context other than in heat-induced PQC foci.

### Gag Foci Localization and Formation Are Interconnected with the sPQC Machinery.

**Fig. 4.**
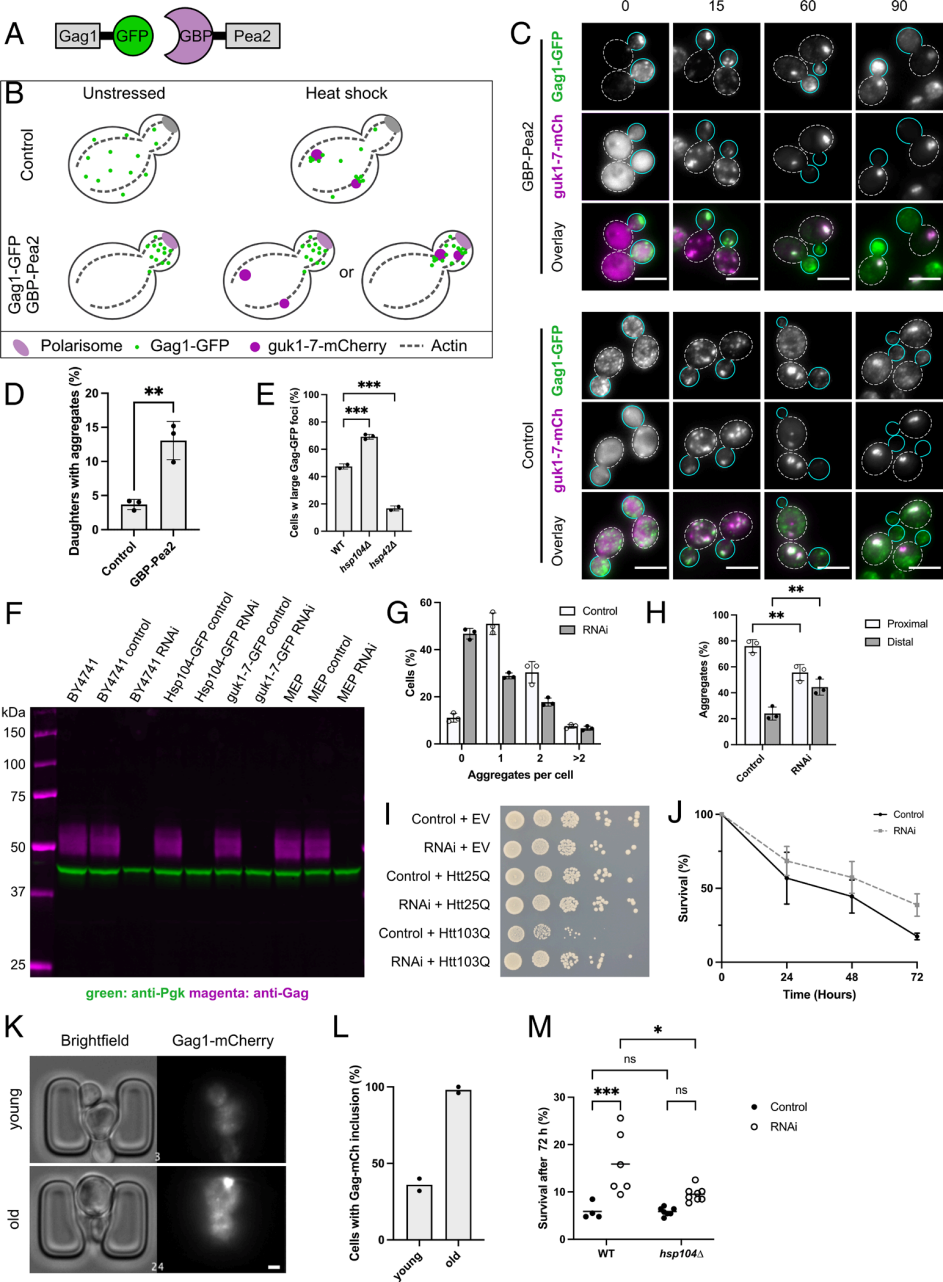
Ty retrotransposons affect spatial PQC, disease protein detoxification, and replicative lifespan. (*A*) Illustration of the Gag transport system. Gag1 is fused to GFP and the GFP-binding protein (GBP) is fused to the polarisome unit Pea2. (*B*) Illustration of the experimental setup for the data in (*C*). In unstressed cells, Gag1-GFP is sequestered to the polarisome into daughter cells by the GBP-Pea2 construct. Upon HS, the aggregates may visibly separate from the Gag clustered in the bud or localize in proximity. (*C*) LM of guk1-7-mCherry and Gag1-GFP expressing cells with GBP-Pea2 or the control construct at indicated time points (min) during continuous HS at 38 °C. Dashed white lines indicate mother cells, and solid cyan lines indicate daughter cells. (Scale bar, 5 µm.) (*D*) Quantification of the fraction of daughter cells with guk1-7-mCherry aggregates of cells shown in (*C*) after 90 min 38 °C HS. (*E*) Quantification of LM of cells with large Gag1-GFP foci at 90 min 38 °C HS in WT, *hsp104*Δ, and *hsp42*Δ. (*F*) Western blot with anti-Gag and anti-Pgk of indicated strains in the exponential growth phase at 30 °C expressing the control or RNAi construct. (*G*) LM quantification of the number of Hsp104-GFP aggregates per cell after 90 min 38 °C HS in RNAi and control construct expressing cells. (*H*) Quantification of LM of Hsp104-GFP aggregate localization relative to mitochondria in RNAi and control cells exposed to 38 °C HS for 90 min. The statistical test is two-way ANOVA with Sidak’s multiple comparison test. (*I*) Spot test of cells containing the control or RNAi construct and expressing empty vector, Htt25Q or Htt103Q plasmids after growth at 30 °C for 3 d. (*J*) Survival over time of MEP cells expressing the control or RNAi construct shown as mean with SEM. (*K*) Microfluidics images of a WT cell expressing Gag1-mCherry at 30 °C. The white number indicates the image frame. Observed budding events at frame 3: 2, frame 24: 16. (Scale bar, 2 µm.) (*L*) Percentage of cells with large Gag1-mCherry inclusion quantified for cells in (*L*). Twenty-five cells each were quantified for young and old cells in two independent replicates. Data points depict the average of 25 cells. (*M*) Survival of MEP cells after 72 h with *hsp104*Δ or with intact *HSP104* (WT) expressing the control or RNAi construct. The line depicts the mean. The statistical test is a two-way ANOVA with a Tukey post hoc test comparing all values. Statistical significance is only indicated for comparisons of interest for clarity.

To determine whether VLPs can influence aggregate localization, we used a synthetic system (46), which sequesters GFP-tagged proteins toward the daughter during cell division due to a fusion of a GFP-binding nanobody (GBP) and Pea2, a polarisome subunit (Fig. 4*A*). We monitored strains containing GBP-linked Pea2 and Gag1-GFP to sequester VLPs into daughter cells, combined with *guk1-7-mCherry* expression during mild HS (Fig. 4 *A*–*C*). Gag1-GFP localized to the bud/daughter cell to a large extent prior to heat stress in GBP-Pea2 cells, as anticipated, a localization that was partially lost at later time points of HS. Gag1-GFP visible in the mother cell appeared generally colocalized with guk1-7-mCherry aggregates. We determined the number of daughters with guk1-7 aggregates and found it to be significantly higher, albeit to a small extent, in the strain expressing GBP-linked Pea2 (Fig. 4*D*), which suggests that VLP movement toward daughters moved a portion of guk1-7 aggregates along with them, potentially because the PQC machinery acts on VLPs. It was previously suggested that there may be a link between molecular chaperones and VLPs (47). We used two established mutants with opposing effects on aggregate formation: *hsp104*∆, which is defective in aggregate sequestration and clearance (7, 8, 48), and *hsp42*∆, which cannot properly form aggregates during stress (8–11). After 90 min at 38 °C, deletion of *hsp104*∆ caused an increase of Gag1-GFP foci compared to wild-type while the *hsp42*∆ mutant had the opposite effect (Fig. 4*E*). It is therefore possible that Hsp42 is required for the formation of large Gag foci in the same way as Hsp42 functions as a sequestrase of protein aggregates. In support of this notion, Hsp42 and Gag foci transiently colocalize during HS (*SI Appendix*, Fig. S4*A*). Alternatively, some Gag1-GFP foci may be bona fide protein aggregates themselves.

### Silencing Retroelement Expression Improves sPQC during Mild HS.

Since deletion of chaperones affected Gag1 foci formation and these foci, in turn, could recruit misfolded proteins, we reasoned that removal of VLPs from the cell may affect PQC. Since Ty genetic elements are spread throughout the genome, they are difficult to target by classic genetics. We therefore searched for a different method to modulate VLP levels and found that RNAi had been shown to silence Gag expression (49). *Saccharomyces cerevisiae* does not have RNAi, but it could be restored by introducing *Saccharomyces castellii* Argonaut and Dicer, which target Ty RNA, yielding *S. cerevisiae* with silenced endogenous retrotransposition (49). We constructed an integrative plasmid containing both Argonaut and Dicer with strong promoters. Strikingly, this construct could efficiently knock down expression of Ty Gag to undetectable levels as seen by Western blot (Fig. 4*F* and *SI Appendix*, Fig. S4*B*). In contrast, introduction of RNAi had no other obvious effects on cellular functions (*SI Appendix*, Fig. S4*C*).

The RNAi strain, along with an empty vector control, were then tested for effects on spatial PQC. During mild HS, we observed an increased removal of Hsp104-GFP labeled aggregates from cells when silencing expression of retroelements (Fig. 4*G* and *SI Appendix*, Fig. S4*D*). Notably, the Hsp104-GFP inclusions were localized distally to mitochondria to a higher extent in the RNAi strain than in WT cells (Fig. 4*H*). This could be due to slower relative clearance of inclusions distal to mitochondria which has been previously observed (5). Accordingly, deletion of *SPT3,* which was found in our screen as a factor involved in aggregate sequestration to mitochondria, and is a known suppressor of Ty function, also showed a decrease in aggregates near mitochondria (*SI Appendix*, Fig. S4*E*). Finally, in further support of positive effects on PQC, we found RNAi to ameliorate toxic effects of a plasmid overexpressing the misfolding Huntingtin exon-1 model protein (Htt103Q, Fig. 4*I*).

### Silencing Ty Retroelement Expression Extends Replicative Lifespan in a Manner Partially Dependent on Fully Functional PQC.

Retrotransposition increases during aging in various cells and organisms, which correlates with an increase in genome instability, a hallmark of aging (50–55). Additionally, old budding yeast mothers form more Ty RNA foci and have increased Gag levels (56). However, the effects of elimination of expression of Ty retroelements on replicative lifespan have not been explored. We used the Mother Enrichment Program (57) to test the effects of the RNAi-mediated silencing of Ty retroelements and observed a two-fold increase in survival at the final time point (72 h, Fig. 4*J*). This effect was reproducible in several backgrounds including those expressing fluorescently tagged Hsp104-GFP (*SI Appendix*, Fig. S4*F*) or the misfolding protein guk1-7-mCherry (*SI Appendix*, Fig. S4*G*). This led us to wonder what happens to VLPs during aging and whether they are subject to spatial PQC. We imaged Gag1-mCherry expressing cells during replicative aging using microfluidics. During aging, large Gag1-mCherry foci appeared (Fig. 4 *K* and *L* and Movie S1) and, in line with previous evidence (56), Gag1-mCherry levels increased (Fig. 4*K* and *SI Appendix*, Fig. S4*H*), suggesting that Gag1-mCherry may behave similarly to age-associated protein aggregates during replicative aging. Elimination of Gag expression and VLPs may be beneficial for cells when they age, e.g., by relieving the PQC machinery of handling Ty Gag proteins and VLPs. In agreement with this possibility, deletion of the general disaggregase *HSP104*, which targets age-associated protein deposits (23, 58, 59), reduced lifespan expansion of the Ty-silenced strain (Fig. 4*M*), suggesting that lifespan extension through silencing of Ty retroelement expression partially depends on a functional PQC machinery. However, when we quantified the numbers of Hsp104-GFP labeled foci found in isolated, replicatively aged cells, there was no noticeable difference between control and RNAi-expressing cells (*SI Appendix*, Fig. S4*I*). Therefore, eliminating endogenous Gag1 accumulation during aging via RNAi does not appear to alter aggregate formation or clearance during aging.

## Discussion

We used immunoelectron microscopy and 3D modeling to show where heat-induced aggregates are deposited in the cell. While aggregates can be found near the nucleus and vacuole as predicted from descriptions of the JUNQ, INQ and IPOD spatial quality control sites, most localized to, or in proximity to, mitochondria and created an area of ribosome exclusion visible by EM. Our unbiased screens for genes required for normal localization of aggegates near mitochondria produced many hits, but these mutations disrupted aggregate localization to at most 20%, in contrast to the ca. 30 to 40% observed with mutants identified in candidate-based approaches (4). Because mitochondria occupy at least 10% of the cellular volume and form expanded networks, there are marginal differences between proximal and distal aggregates. Quantification methods will therefore highly influence these types of data (60).

From our high-content microscopy screen hits, we decided to further characterize *CLU1* since it was not previously described to influence aggregate deposition, it had a known role in mitochondrial function and because it did not cause any marked defects in aggregate formation or coalescence. While *clu1*Δ cells have clustered mitochondria, abnormal mitochondrial morphology is not necessarily a cause of aggregate mislocalization, as other mutants with similar morphology phenotypes did not result in different patterns of aggregate localization. Interestingly, disruption of this seemingly central aggregate deposition site that spatial PQC pathways of various protein species converge on, did not cause major defects in protein aggregation behavior or cellular fitness. In fact, we observed no major deleterious consequences directly linked to a change in aggregate localization. However, since localization of aggregates near mitochondria in the mutants identified was only disrupted to a limited extent, any striking effects on PQC could be masked or too small to discern. A recent publication identified the mitochondrial outer surface receptor Tom70 as a key component contributing to the localization of cytosolic protein aggregates to mitochondria via multivalent hydrophobic interactions (61). Our high-content microscopy screen could not identify *tom70*Δ as a mutant with disrupted localization of aggregates to mitochondria since we select for Tom70-mRuby2 during the SGA crossing procedures.

3D models of EM images revealed assembled VLPs clustering at the mitochondrial protein deposition site during HS. Assembly of VLPs represents a key step in the life cycle of retrotransposons and Ty transposable elements share many similarities with retroviruses (36, 62–64). The Ty transposons of yeast are continuously expressed and subsequently result in continuous assembly of VLPs. The VLPs can be delivered to the nucleus to insert additional Ty cDNA into the genome, thereby completing retrotransposition. Environmental changes such as low temperature and nitrogen starvation cause an increase in retrotransposition frequency; however, retrotransposition events of Ty1 and Ty3 are drastically reduced at elevated temperature (65–69). We therefore do not consider retrotransposition to have a major impact on our observations at 30 °C or higher. One possibility is that VLPs cluster in the same location as aberrant proteins in an attempt to minimize crowding and shield VLPs from the cellular environment. Alternatively, they could be subject to degradation in a pathway similar to misfolded proteins. A recent study also identified a prion-like domain in the Ty1 Gag protein, which is responsible for VLP assembly and thus appears implicated in phase separation processes (70). Our data suggest that VLPs interact with PQC components based on three lines of results. First, colocalization by both light and electron microscopy. Second, artificial relocation of a VLP component influenced the localization (sequestration) of an unrelated misfolding protein. Third, removal of Gag/VLPs improved spatial PQC in that aggregate clearance was enhanced and the toxicity of a Huntingtin disease model protein reduced. It is possible that VLPs interfere with sPQC function directly or that processing of Gag/VLPs causes an increased burden on the PQC machinery, especially during stress and aging. In addition, replicative lifespan extension by RNAi-silencing of Ty expression partially depended on Hsp104 function, further linking VLPs to effects on PQC. One interpretation of this is that removal of VLPs extends replicative lifespan by both relieving a burden on the PQC system and by acting through a different pathway, such as by reducing genomic instability that normally correlates with increased retrotransposition and chromosomal rearrangements with age (56).

Ty elements make up 3 to 4% of the *S. cerevisiae* genome and we show here that continuous expression can put a burden on the PQC machinery. It has been estimated that human endogenous retroviruses make up 4 to 8% of the human genome (71). They have also been shown to be expressed in several cell lineages, including stem cells (72). It is therefore possible that expression of these endogenous retroviruses could have similar detrimental effects on human cells, especially during aging. In fact, a recent publication identified retrovirus-like particles of human endogenous retroelements as drivers of aging and their repression alleviated phenotypes associated with senescence (21). Our finding that silencing of retroelements could reduce the toxicity of a model of Huntington’s disease raises the question whether stem cell aging could be affected in a way similar to replicatively aged yeast. Our study therefore highlights the importance of potential consequences of retroelement expression aside from retrotransposition.

## Materials and Methods

### Yeast Media, Plasmids, and Strains.

*S. cerevisiae* strains were cultured in rich media (YPD, 2% dextrose) at 30 °C according to standard protocols. Temperature-sensitive strains were grown at 22 °C. Strains containing plasmids that require selection for maintenance were cultured in the appropriate drop-out medium. The strain background was BY4741 (S288C) except for imaging screens (SGA background). Strain genotypes, plasmids, and origins are listed in *SI Appendix*, Table S1. Refer to *SI Appendix* for detailed strain constructions.

To create fluorescently tagged Gag, the first 1203 bp of ORF YER137C-A was cloned into a vector containing a 3′ GFP in frame as well as a GPD (*TDH3*) promoter to make pPW427. This results in continuous expression of the first 401 amino acids of a Ty1 Gag, Gag-p45, fused to GFP. This 45-kD capsid protein assembles into VLPs (36, 73). The Gag gene fragment was subcloned into a vector containing a C-terminal mCherry in frame to make pPW435. Strains containing fluorescently tagged guk1-7, gus1-3, and pro3-1 misfolding proteins were created as described previously (27). mRuby2-tagged versions were created by first subcloning each of them into a pRS405 vector containing a LYS fragment for integration. The GFP was then replaced with mRuby2 amplified from pFA6a-link-yomRuby2-Kan (74), a gift from Wendell Lim (Dept. of Cellular and Molecular Pharmacology, UCSF) and Kurt Thorn (Nikon Imaging Center, UCSF) via Addgene (#44953), to create plasmids pPW388, pPW389, and pPW390. The Tom70-mRuby2 mitochondrial marker was generated as described in ref. 5. Tom70-mScI was constructed as in ref. 75. Deletion mutants, C-terminal protein fusions with GFP, and overexpression strains were constructed using pYM plasmids and protocols from ref. 76 or stem from yeast collections. The Huntingtin exon plasmids pRS416-GPD-Htt25Q-GFP and pRS416-GPD-Htt103Q-GFP were gifts from Susan Lindquist via Addgene (#1177, #1180).

GBP-Pea2 strain construction: Plasmid and strain constructions are described in ref. 46 and in *SI Appendix*.

### Light Microscopy and Data Analysis.

Light microscopy was performed with a conventional fluorescence microscope, the Zeiss Axio Observer .Z1 inverted microscope equipped with an Axiocam 506 m camera and a Plan-Apochromat 100x/1.4 NA Oil DIC M27 objective. Images were acquired with Z-stacks except where indicated and manually quantified with the help of the CellCounter Plugin in Fiji (ImageJ). Data were mainly used to calculate the fraction of cells with protein aggregates/Gag1 foci or the fraction of cells containing ≥three aggregates per cell. ≥200 cells were counted for each condition, strain, and replicate. To assess localization of aggregates relative to mitochondria, the initial quantification was in two categories, proximal and distal aggregates to Tom70 signal, which was later adjusted for clarity by classifying three categories of localization, colocalized (complete or partial overlap of signal), proximal, and distal. ≥200 aggregates were counted for each condition, replicate, and strain. Results are displayed for simplicity without including category proximal or by pooling proximal and distal or colocalized and proximal into one category. Sixty- and 90-min time points were used for quantifications because they showed the clearest localization of aggregates. Aggregate asymmetry was assessed by manually determining daughter cells/buds with aggregates among mothers with aggregates at time point 90 min. ≥200 budding events were counted for each condition, strain, and replicate. Representative images of microscopy experiments are shown in maximum Z-projection of relevant Z-steps or single Z-planes. Brightness/contrast adjustments were made to highlight colocalization.

Images of immunofluorescence with anti-Hsp42 and anti-Gag were deconvolved using the Diffraction PSF 3D and Iterative Deconvolve 3D plugins in Fiji as described in ref. 77. The deconvolved Z-stacks were maximum projected. Brightness/contrast was adjusted to visualize colocalization between Gag1-GFP and anti-Hsp42. Brightness/contrast was adjusted to the same values for each image of the anti-Gag time course.

### Hsp104 Interaction Data.

Data were extracted from those published in ref. 39. Unweighted spectrum counts of all hits were compared to peptide counts in ProteinAtlas for *S. cerevisiae*. Statistical significance was calculated using a Fisher’s exact test. Spectral counts for both controls and Hsp104-GFP co-IPs are in columns D, E and F, G respectively. Column H shows the number of observed peptides for the listed protein in the database. Column I shows the total peptides observed in the Peptide atlas for the positive hits in each column D-F. Column J shows the number of peptides observed in each mass spectrometry run. K is relative abundance, and column L is the statistical significance.

### Electron Microscopy Sampling, Immunolabeling, and 3D Modeling.

Sample preparation and electron microscopy were performed as described previously (16). Immunogold labeling was performed according to previous protocols (27), with a rabbit anti-GFP primary (1/5 dilution when identifying guk1-7-GFP and 1/30 when identifying Hsp104-GFP, ab6556, Abcam), followed by detection with the secondary antibody, 10 nm gold goat anti-rabbit (1/20 dilution, EMS). For 3D reconstruction, serial 70-nm-thin sections were immuno-labeled and imaged. The resulting micrographs were then aligned and analyzed with IMOD (78) to create 3D reconstructions of organelles and the localization of the gold particles to visualize protein aggregates.

### Quantification of EM Data.

Measurements of VLPs and cluster areas were performed by manually tracing clusters or by modeling VLPs through application of an elliptical shape. 3D models were done like above, using serial sections and IMOD software.

In micrographs or 3D models with electron microscopy without quantification, we used earlier HS time points to ensure that more protein aggregates per cell are visible across the cell to acquire sufficient numbers in thin 70-nm sections. We used time points 60 or 90 min to quantify the formation of larger inclusions after smaller Q-bodies have coalesced. For quantification of protein aggregate proximity to specific cellular structures, the closest organelle to which the aggregate located was scored in thin-section electron micrographs. If an aggregate localized equidistant to two organelles, each organelle was scored once. Cytosolic refers to protein aggregates not in direct proximity to any organelle.

Analysis of immunogold labeling against HSP104-GFP was performed by marking individual gold particles and the area of the respective structure. Then, the gold labeling density was determined in the number of gold particles per µm^2^.

To determine clustering of VLPs, over 320 cell sections were counted, resulting in a total 2,200 counted VLPs. VLPs were considered a cluster if at least two VLPs were in close proximity with no ribosomes between them.

### High-Content Imaging Screen.

SGA mating to introduce gus1-3-GFP Tom70-mRuby2 and Hsp104-GFP and Tom70-mRuby2 into the mutant collections was performed according to published protocols (28). The imaging screen was performed in 96-well format. Cells were grown to mid-log phase in YPD at 30/22 °C, shifted to 38 °C for 90 min (deletion library) or 120 min (ts library), fixed with 3.7% formaldehyde (final concentration), and washed with PBS. Imaging was performed in glass bottom plates (Matriplate) using the ImageXPress Micro XLS (Molecular Devices) high-content microscope equipped with a 100× objective (CFI L Plan EPI cc 0 mm to 0.7 mm). Acquired images were segmented and quantified using MetaXpress. The output used to sort mutants was the fraction of colocalized aggregates normalized to the average of the control cells (*his3*∆) on the same plate, resulting in a value for deviation from WT value. Only strains with ≥50 cells to analyze in the images were considered, and all hits with “dubious ORF” annotation on SGD were removed. The remaining top hits were analyzed for functional enrichment using Spatial Analysis of Functional Enrichment [SAFE (29) in https://thecellmap.org/, *P* ≤ 0.05 (79)] and DAVID GO term enrichments (https://david.ncifcrf.gov) applying default settings using the GOTERM_BP_DIRECT option with a *P*-value < 0.05. The respective GO term analysis was performed using the complete deletion or ts allele library as background. Only enriched pathways with at least five genes were considered.

### Mother Enrichment Program to Monitor Lifespan.

PWY1422 (control) and PWY1423 (RNAi) were inoculated into YPD medium, grown overnight, diluted, and regrown to OD_600_ = 0.5. Cultures were diluted to approximately 2000 CFU/mL, and 100 µL of each was plated on YPD agar plates to be counted after 2 d. Estradiol was added to each culture to 1 µM final and split into 20 mL cultures. CFU was counted every 24 h by taking 100 µL and washing 1x with 1 mL YPD before plating on YPD agar. At least 3 cultures that did not acquire an “escape” mutation were used to construct a survival plot. Survival over time is visualized as mean with SEM error bars. PWY1430 and PWY1431 were treated similarly, except 10 x 3 mL cultures containing approximately 5,000 CFU/mL were used to test each strain. Escape mutants were eliminated if they met either of the following criteria: 1) Cultures had a higher CFU than the starting culture 2) They had a CFU count greater than three SDs of the mean. To further lower the frequency of an escape mutant in an individual culture, 10 x 2 mL cultures at approximately 5,000 CFU/mL were used for all other MEP aging experiments.

### Isolation of Replicatively Aged Cells.

The experiment was performed as described previously via the established MagnaBind biotin–streptavidin method (80). Biotin-labeled cells were isolated after growth overnight until OD < 1. The young cell control was acquired from unbound cells during the first magnetic sorting step.

## Supplementary Material

Appendix 01 (PDF)

Dataset S01 (XLSX)

Dataset S02 (XLSX)

Dataset S03 (XLSX)

Dataset S04 (XLSX)

Dataset S05 (XLSX)

Dataset S06 (XLSX)

Dataset S07 (XLSX)

Movie S1. **Microfluidics time-lapse microscopy of WT cells expressing Gag1-mCherry.** Exponentially growing cells trapped in a chip were monitored during their replicative lifespan at 30°C by imaging once every hour for 25 hours. The movie shows brightfield and Gag1-mCherry (red) channels as in Figure 4K. Contrast/brightness were adjusted to the same levels throughout the movie for visualization.

## Data Availability

Previously published data were used for this work (39). All other data are included in the manuscript and/or supporting information.
