# Supplementary material for: Elimination of virus-like particles reduces protein aggregation and extends replicative lifespan in Saccharomyces cerevisiae
Source: Proc Natl Acad Sci U S A. 2024 Mar 25;121(14):e2313538121. doi: 10.1073/pnas.2313538121 (PMC10998562; doi:10.1073/pnas.2313538121)
Supplement: Supplementary file 1 — Appendix 01 (PDF) [file pnas.2313538121.sapp.pdf]

# Supplementary figure 1

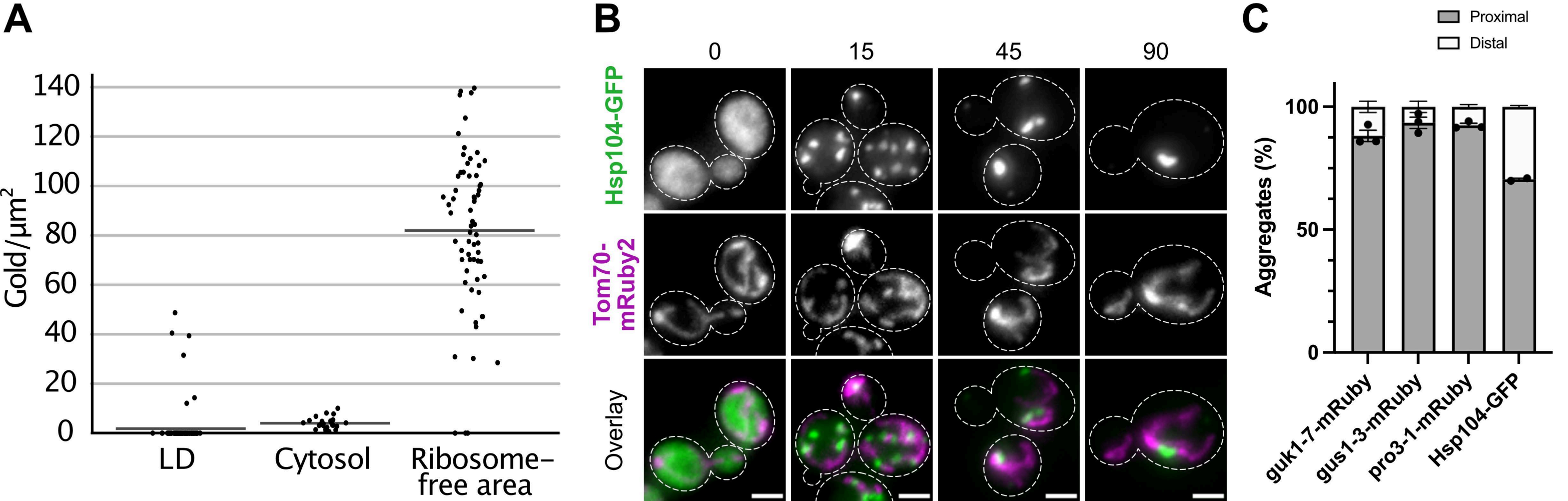

# Supplementary figure 2

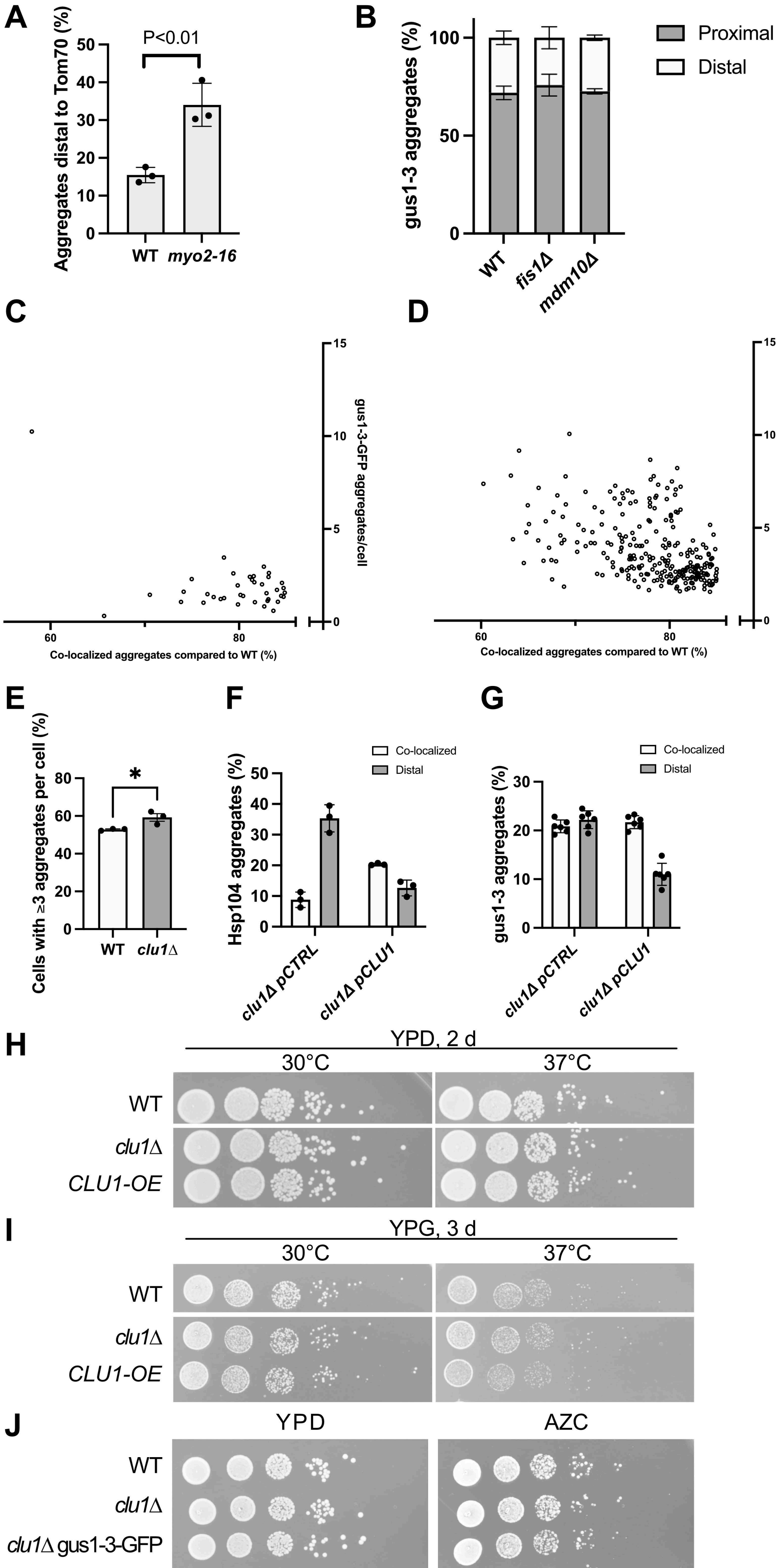

## Supplementary figure 3

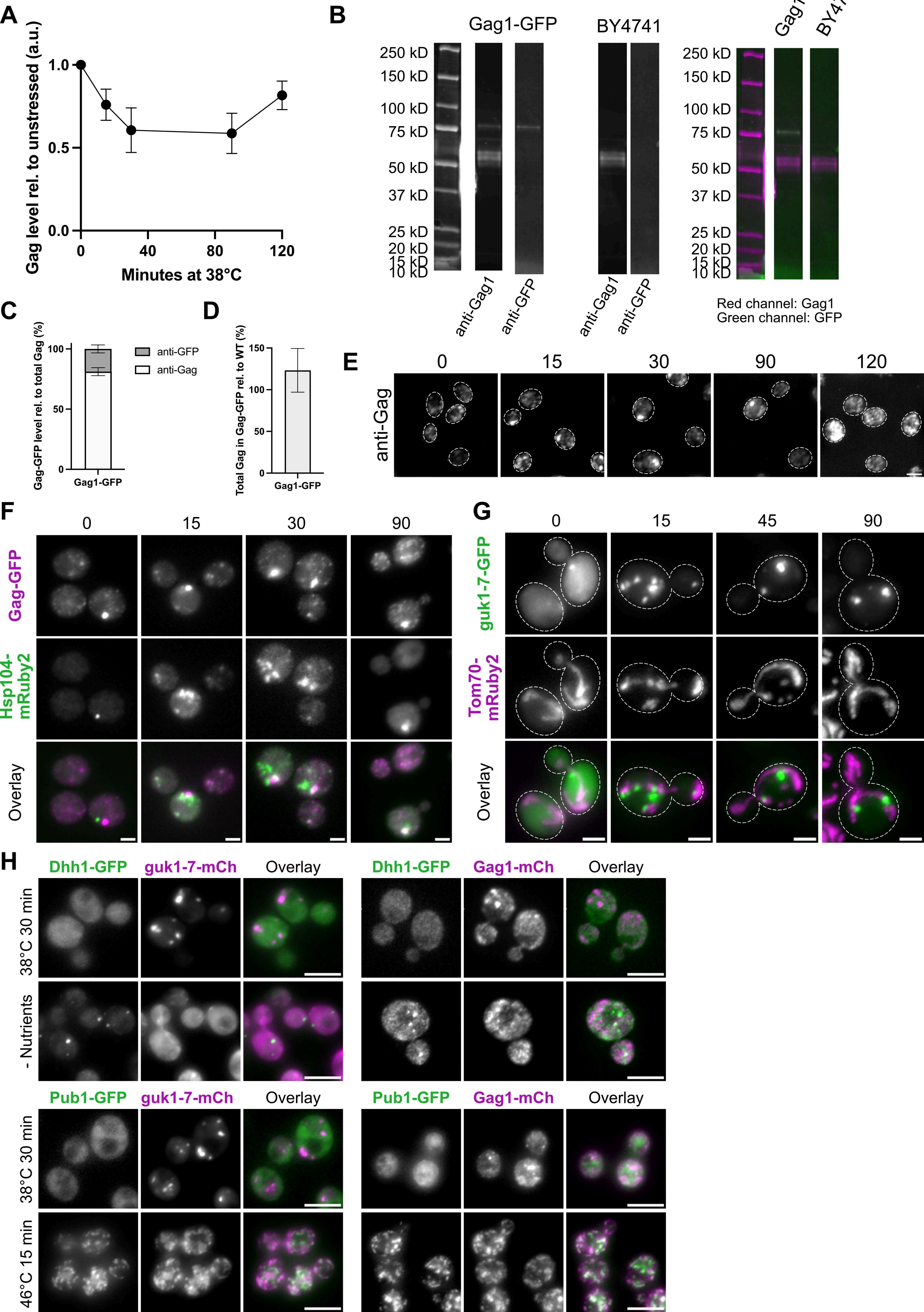

# Supplementary figure 4

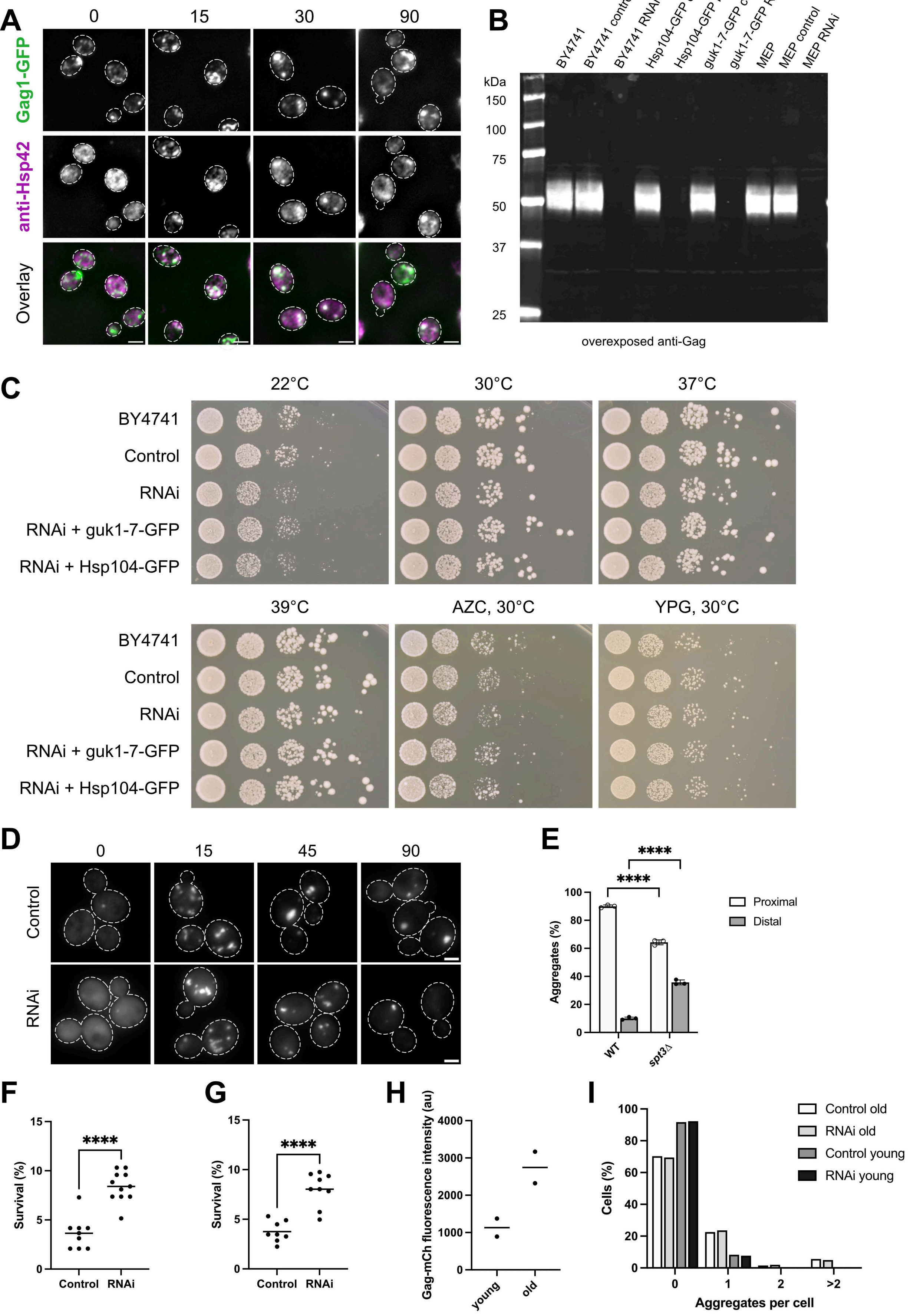

## Supplementary Figure Legends

**Supplementary figure 1: Protein aggregates of various protein species are often in close proximity to mitochondria.** A) Gold labeling density of indicated structures in Hsp104-GFP expressing cells subjected to 90 min of HS and imaged with immuno-EM. Gray line=mean labeling density, n=79 cells. B) LM images showing cells expressing Hsp104-GFP and the mitochondrial marker Tom70-mRuby2 exposed to continuous mild heat shock at 38°C for the indicated time in minutes. Scale bar 2 $\mu$m. C) Quantification of aggregate localization relative to mitochondria in cells exposed to 38°C HS for 90 min and imaged with fluorescent microscopy. Aggregates of the different reporters were scored as proximal or distal to mitochondria.

**Supplementary figure 2: Lack of Clu1 impairs aggregate localization to mitochondria but has no effects on fitness upon proteotoxic stress**. A) Fraction of Hsp104-GFP aggregates distal to mitochondria in WT and *myo2-16* cells after 90 min HS at 38°C. B) Quantification of gus1-3-GFP aggregate localization relative to mitochondria after 90 min HS at 38°C in WT and *fis1Δ* and *mdm10Δ* cells. C) Scatter plot of number of gus1-3-GFP aggregates per cell over aggregate co-localization compared to WT of the hits found among the ts allele collection. D) As C but for Hsp104-GFP. E) Fraction of cells with ≥3 gus1-3 aggregates/cell in WT and *clu1*Δ cells determined after 60 min HS at 38°C. F) Complementation of Hsp104-GFP aggregate localization relative to mitochondria after 60 min HS at 38°C. G) Complementation of gus1-3-GFP aggregate localization as in 2F. H) Fitness of *CLU1* mutant strains at indicated temperatures after growth for depicted number of days (d) on YPD. I) As H but on YPG (3% glycerol). J) Fitness of *clu1*Δ and *clu1*Δ cells expressing gus1-3-GFP on YPD compared to YPD + AZC (0.6 mg/ml) at 30°C.

**Supplementary figure 3: Heat-induced protein aggregates are often in close proximity to mitochondria and VLPs.** A) Quantification of Ty Gag levels at indicated time points in BY4741 cells during continuous heat shock at 38°C. Gag levels were normalized to Pgk1 levels and time point 0 Gag level was set to 1. Plot shows mean and SEM of 4 replicates. B) Western blot with anti-Gag recognizing endogenous Ty Gag and Gag1-GFP in lysates of logarithmic phase cells of BY4741 and Gag1-GFP expressing cells at 30°C. C) Quantification of endogenous Ty Gag and Gag1-GFP levels in lysates of logarithmic phase cells of Gag1-GFP cells at 30°C. D) Western blot quantification as in C but showing total Gag levels in Gag1-GFP cells relative to WT cells. E) Immunofluorescence with anti-Gag of BY4741 during a time course at 38°C HS. Scale bar 2 $\mu$m. F) LM of Hsp104-mRuby2 and Gag1-GFP expressing cells at indicated time points (min) during continuous heat shock at 38°C. Scale bar 2 $\mu$m. G) LM of Tom70-mRuby2 and guk1-7-GFP expressing cells at indicated time points (min) during continuous heat shock at 38°C. Scale bar 2 $\mu$m. (H) LM of Dhh1-GFP and Pub1-GFP each combined with expression of guk1-7-mCherry or Gag1-mCherry exposed to indicated stress conditions. Scale bar 5 $\mu$m.

**Supplementary figure 4: The interplay between VLPs, PQC and replicative lifespan.** A) Immunofluorescence with anti-Hsp42 of a time course during 38°C HS of Gag1-GFP expressing cells. Scale bar 2 $\mu$m. B) Overexposed image of blot in 4F to visualize anti-Gag detection. C) Spot test of BY4741 and BY4741 expressing the control or RNAi construct and cells expressing the RNAi construct combined with

either guk1-7-GFP or Hsp104-GFP. Cells were grown at indicated temperatures on YPD, YPD + AZC (0.6 mg/ml) or YPG (3% glycerol) for 3 days. D) LM of Hsp104-GFP (grayscale) and Tom70-mScI (not imaged) expressing cells without or with active RNAi at indicated time points (min) during continuous heat shock at 38°C. Scale bar 2 $\mu$m. E) Quantification of Hsp104-GFP aggregate localization relative to mitochondria of WT and *spt3Δ* cells after 90 mint 38°C HS. Statistical test is 2-way ANOVA with Sidak's multiple comparison test. F) Survival of MEP cells with Hsp104-GFP fusion after 72h expressing the control or RNAi construct. The line depicts the mean. G) As in F, for cells containing guk1-7-mCherry. One data point was excluded from the control since it was considered an outlier value at ≥2.5 SD above the mean. H) Gag1-mCherry fluorescence intensity in young and old cells of the microfluidics data shown in 4K. Line depicts the mean. I) Quantification of Hsp104-GFP aggregates in replicatively aged cells expressing the control or RNAi construct (n=200, N=1).

**Supplementary movie: Microfluidics time-lapse microscopy of WT cells expressing Gag1-mCherry.** Exponentially growing cells trapped in a chip were monitored during their replicative lifespan at 30°C by imaging once every hour for 25 hours. The movie shows brightfield and Gag1-mCherry (red) channels as in Figure 4K. Contrast/brightness were adjusted to the same levels throughout the movie for visualization.

## SUPPLEMENTARY METHODS

### CLU1 strain construction
The genome-integrative plasmid containing *CLU1* under its native promoter was created by amplifying the *CLU1* ORF plus 505 bp upstream and 500 bp downstream with EcoRV and AscI restriction sites added and ligating into pPW411 to create pPW425.

### SGA query strain construction
A genome-integrative plasmid containing the C-terminal region of *TOM70* followed by *mRuby2* and *gus1-3-GFP* was integrated in the SGA query strain Y7092 for high content microscopy screening. This plasmid was created by amplifying *mRuby2* from *TOM70-mRuby2* with overhang of the C-terminal region of *TOM70* from our previously created construct and inserting it into plasmid pPW374 with PacI/NsiI. pPW374 contains gus1-3-GFP with resistance to ClonNat. The final plasmid was sequenced in relevant regions and integrated in the C-terminal region of the *TOM70* locus, thereby inserting mRuby2 and gus1-3-GFP simultaneously.

### RNAi strain construction
pRS405-PTEF-Dcr1 and pRS404-PTEF-Ago1 were gifts from David Bartel via Addgene (#22314, #22313). The AGO1 ORF was cut out of pRS404-PTEF-Ago1 using SpeI/XhoI and subcloned into pPW358 cut with XbaI/SalI, to create pPW452, putting AGO1 between a GPD promoter and PGK1 terminator. DCR1 with TEF promoter and CYC1 terminator was cut from pRS405-PTEF-Dcr1 using SacI/BsiEI and ligated into pPW452 cut with SacI/SacII to create pPW455. pPW455 was cut with SacI/ApaI to move AGO1 and DCR1 with promoter and terminator sequences into pPW454, a vector with HPH (hygromycin resistance) flanked by 5’ and 3’ sequences of the *URA3* ORF, to create pPW456. pPW454 and pPW456 were cut with PmeI to linearize and were subsequently transformed into BY4741 to create PWY1420 (control) and PWY1417 (RNAi), respectively.

### MEP strain construction
pPW456 was cut with SacI/ApaI and the AGO1/DCR1 fragment ligated to pPW459 digested with SacI/ApaI to create pPW460. pPW459 contains *S. pombe HIS5* flanked by sequences 5’ and 3’ of the HIS3 ORF. pPW459 and pPW460 were cut with PmeI to linearize and were subsequently transformed into the Mother Enrichment Program strain UCC5179 to create PWY1422 (control) and PWY1423 (RNAi), respectively. PWY1422 and PWY1423 were transformed with a GFP-KanMX cassette targeting the C-terminus of Hsp104 to create PWY1426 and PW1427. They were also separately transformed with a PCR-amplified *HSP104* deletion cassette with KanMX to create PWY1430 and PWY1431.

### GBP-Pea2 strain construction
The genes for the GFP-binding protein (GBP) and Pea2 were PCR amplified and combined using Gibson Assembly (NEB) and integrated into pPW411, which had a *KanMX* cassette instead of a *NatMX* cassette, yielding pAF118. This plasmid was genomically integrated at the *MET15* locus, by cutting the plasmid with PmeI (ThermoFisher Scientific), which introduces two cuts and exposes homology regions

to the *MET15* locus. The integrated cassette contained the *CYC1* terminator and the weak constitutive *ADH1* promoter.

### VLP antibody, protein extraction, Western blot und quantification

Cells were harvested from mid-logarithmic cultures ($OD_{600}$~0.5) and re-suspended in 0.2 M NaOH. After 5 min incubation at RT, cells were pelleted and re-suspended in 50 $\mu$l of standard 2x sample buffer (Laemmli), followed by 3 min incubation at 98°C. Supernatant was loaded on precast Criterion TGX 4–12% or 10% acrylamide gradient gels (Bio-rad). Proteins were separated and then transferred to PVDF membranes for detection using a wet tank blotting system (Criterion blotter, Bio-rad). Membranes were blocked for 30 min with Odyssey blocking buffer (PBS, LI-COR) at RT, followed by incubation with primary antibodies specific for the Gag1 N-terminus (rabbit, VTSKEVHTNQDPLD, 1/5000) and Pgk (mouse, 22C5D8, Invitrogen, 1/5000). Secondary antibodies (goat anti-mouse IRDye 800CW and goat anti-rabbit IRDye 680, LI-COR, 1/10000) were incubated for 1h at RT and fluorescence detected using the Odyssey Infrared scanner. Signal was quantified using Fiji (ImageJ) by normalizing to Pgk1 signal as a loading control.

### Heat stress and nutrient removal

Cells were grown to mid-logarithmic phase ($OD_{600}$~0.5) at 30°C and subjected to continuous mild heat shock at 38°C and fixed in 3.7% formaldehyde (final concentration) at indicated time points or imaged directly. For severe heat shock to visualize stress granules, cells were shifted to 46°C for 15 min before imaging. To induce visible P-body formation, cells were harvested, re-suspended in water and incubated 30 min at room temperature.

### Serial growth assay

Cells were grown to mid-logarithmic phase in YPD, washed with water and diluted to $OD_{600}$=0.5 in YP followed by 10-fold serial dilutions, of which 5 $\mu$l were spotted onto YPD, YPD+AZC (0.6 mg/ml) or YPG (3% glycerol) plates. Plates were incubated at indicated temperature and imaged after 2 days (YPD) or 3 days (YPG) of growth. Cells expressing pRS416 plasmids were grown in -Ura drop out media supplemented with 2% glucose to mid-logarithmic phase, their $OD_{600}$ adjusted to 0.5, spotted as described and imaged after 3 days of growth at 30°C on -Ura drop out plates.

### Immunofluorescence

BY4741 cells were grown to $OD_{600}$~0.5 in YPD, shifted to 38°C and harvested at indicated time points for fixation. Cells were treated as described previously (26) with the modification that permeabilization and blocking buffers contained 0.5% BSA. Primary antibody incubation was performed with block solution containing rabbit anti-Gag1 antibody (1/2500, VTSKEVHTNQDPLD, Genscript) at 4°C ON. Slides were washed and incubated with secondary antibody (goat anti-rabbit AF568, Invitrogen, 1/500 in block solution) for 1h at RT and subsequently stained with 1 $\mu$g/ml DAPI 0.1%

Triton X-11 for 10 min at RT. Finally, mounting media (ProLong Glass Hard-set Antifade Mountant, Thermo) was added and slides were sealed with nail polish.

For anti-Hsp42 immunofluorescence, exponentially growing Gag1-GFP cells were subjected to heat stress at 38°C and harvested at indicated time points for fixation. The staining protocol was performed as described previously (26). To avoid unspecific reactions of rabbit anti-Hsp42 antibody, the antibody was incubated with membrane containing lysate from *hsp42Δ* cells before applying it to immunofluorescence. Primary antibody incubation was then performed with block solution containing rabbit anti-Hsp42 antibody (1/2000, gift from Prof. Johannes Buchner) at 4°C ON. Slides were washed and incubated with secondary antibody (goat anti-rabbit AF568, Invitrogen, 1/500 in block solution) for 1h at RT and subsequently stained with 1 $\mu$g/ml DAPI 0.1% Triton X-11 for 10 min at RT.

## Statistical analyses

Fluorescence images were quantified by manually scoring ≥200 cells per replicate and using the population average as one individual data point. Each replicate is indicated by individual data points in bar graphs. Bars depict the average of all replicates combined and error bars are standard deviation unless indicated otherwise. Statistical testing of two populations compared with one variable was performed using unpaired two-tailed t-test. Comparison across more groups than two was based on One-way ANOVA with multiple comparisons to one control group (WT). These descriptions apply to all data except when indicated otherwise. Data visualization and statistical tests were achieved with GraphPad Prism software. Asterisks above groups denote statistical significance determined by the indicated statistical test with ns $p>0.05$, * $p\leq0.05$, ** $p\leq0.01$, *** $p\leq0.001$, **** $p<0.0001$.

## Microfluidics time-lapse microscopy

Microfluidics time-lapse microscopy were performed using a Zeiss Axio Observer .Z1 inverted fluorescence microscope with Definite Focus, equipped with a Axiocam 506 mono camera (Zeiss). Images were taken with a 100X Plan-APOCHROMAT oil immersion objective (NA 1.4). During the experiment, the microfluidics device (iBiochips) was heated to 30°C in a heating chamber. The microscope was programmed to acquire images every 60 min for a total of about 65 hours. Camera binning was set to 3. A stack of three planes was taken. Cells were grown overnight in SD medium (prepared from CSM powder from Formedium with 2% glucose). The next day, cells were diluted to an $OD_{600}$ of 0.1 and grown to an OD of 0.6. Cells were put into a 1 ml syringe and four yeast strains were loaded at the same time into the microfluidics chip using a NE-4000 syringe pump (New Era Pumps) with a flow rate of 1 $\mu$l/min. As soon as the traps were filled, the cell loading ports were closed with a metal pin. The medium flow rate was set to 10 $\mu$l/min at the beginning using a NE-1000 syringe pump (New Era Pumps). On the next day 12 $\mu$l/min and on the last day 15 $\mu$l/min were used. Sterile filtered SD medium in a 60-ml syringe was used. Time-lapse images were analyzed manually, and the time point of a phenotypic change was registered. Amount of cell divisions were counted after a phenotypic change. The analysis was referenced to the last successful cell division, because this a precise, singular time point and easy to determine in contrast to the exact time point of cell

death. Categories “young” or “old” correspond to cells in the first image frame or after at least 15 divisions, respectively.
